# An AI-based patient-specific clinical decision support system for OA patients choosing surgery or not: study protocol for a single-centre, parallel-group, non-inferiority randomised controlled trial

**DOI:** 10.1186/s13063-022-07039-5

**Published:** 2023-01-12

**Authors:** Nanna Kastrup, Helene H. Bjerregaard, Mogens Laursen, Jan B. Valentin, Søren P. Johnsen, Cathrine E. Jensen

**Affiliations:** 1 Danish Center for Healthcare Improvements, Aalborg University, Frederik Bajers Vej 5, 9220 Aalborg, Denmark; 2 The Danish Clinical Quality Program – National Clinical Registries, Hedeager 3, 8200 Aarhus N, Denmark; 3 Orthopaedic Department, Aalborg University Hospital, Hobrovej 18-22, DK-9000 Aalborg, Denmark; 4 Danish Center for Clinical Health Services Research, Aalborg University, Frederik Bajers Vej 5, 9220 Aalborg, Denmark

**Keywords:** Artificial intelligence, Machine learning, Randomised controlled trial, Cost-effectiveness, Clinical decision support system, Total hip replacement, Total knee replacement, Osteoarthritis, Patient-reported outcomes

## Abstract

**Background:**

Osteoarthritis (OA) affects 20% of the adult Danish population, and the financial burden to society amounts to DKK 4.6 billion annually. Research suggests that up to 75% of surgical patients could have postponed an operation and managed with physical training. ERVIN.2 is an artificial intelligence (AI)-based clinical support system that addresses this problem by enhancing patient involvement in decisions concerning surgical knee and hip replacement. However, the clinical outcomes and cost-effectiveness of using such a system are scantily documented.

**Objective:**

The primary objective is to investigate whether the usual care is non-inferior to ERVIN.2 supported care. The second objective is to determine if ERVIN.2 enhances clinical decision support and whether ERVIN.2 supported care is cost-effective.

**Methods:**

This study used a single-centre, non-inferiority, randomised controlled in a two-arm parallel-group design. The study will be reported in compliance with CONSORT guidelines. The control group receives the usual care. As an add-on, the intervention group have access to baseline scores and predicted Oxford hip/knee scores and HRQoL for both the surgical and the non-surgical trajectory. A cost-utility analysis will be conducted alongside the trial using a hospital perspective, a 1-year time horizon and effects estimated using EQ-5D-3L. Results will be presented as cost per QALY gain.

**Discussion:**

This study will bring knowledge about whether ERVIN.2 enhances clinical decision support, clinical effects, and cost-effectiveness of the AI system. The study design will not allow for the blinding of surgeons.

**Trial registration:**

ClinicalTrials.gov NCT04332055. Registered on 2 April 2020.

## Background

Osteoarthritis (OA) is a major public health concern worldwide. A global burden of disease study of OA showed that cases increased 113.25% from 247.51 million in 1990 to 527.81 million in 2019 [1]. Anatomic, site-specific, age-standardised prevalence rates have increased for knee and hip OA, and the estimated annual percentage changes are 0.32 and 0.28, respectively [1]. Osteoarthritis is associated with pain, disability and loss of function for patients; therefore, healthcare providers and policymakers should be aware of the increasing burden of the disease [1]. International clinical guidelines for the treatment of OA recommend physical training and complementary pharmacological treatment as first-line treatment modalities, and when these options no longer have a positive effect, assessment for joint replacement surgery is advised [2]. In 2014, more than 370,000 primary total hip replacements (THR) were undertaken in the USA and 37,000 were conducted in Australia and 97,000 in the UK in 2017 [3]. In 2008, the USA and Australia conducted 6,485,000 and 359,000 total knee replacements (TKR), respectively, and the UK performed 793,000 surgeries in 2009 [4]. In Denmark, OA affects 20% of the adult population with an economic burden to society of DKK 4.6 billion per year [5, 6]. Although pain symptoms can be managed with pharmaceuticals, and physical training may slow disease progression, nearly 11,000 and 10,500 hip and knee replacements are performed annually in Denmark [2, 7, 8].

However, for all the mentioned treatment options, the guidelines do not provide clear guidance about when surgery is most beneficial for the individual patient. Consequently, under or over utilisation of healthcare resources may occur [2, 9–11]. Research suggests that up to 75% of patients could have postponed surgery and managed with physical training instead [12]. Additionally, matching patients’ expectations with achievable changes in function level can be challenging, which might help explain why up to 28% of the patients who undergo THR or TKR are dissatisfied post-surgery [13, 14]. The limited time to provide tailored treatment options and the adjustment of patients’ unrealistic expectations both prompt the high proportion of dissatisfied patients [13]. Providing access to predicted patient-specific outcomes, including health-related quality of life (HRQoL) consequences, before surgery may be important to improve decision-making and patient satisfaction. One solution to provide this information could be artificial intelligence (AI).

ERVIN.2 is an AI-based clinical decision support system designed to support patient involvement in decisions concerning knee and hip replacement. The system allows for the patient and surgeon to compare real-time (the reported scores on the day of consultation) and one-year prediction scores, negative or positive, in function and pain (OKS/OHS) and HRQoL (EQ-5D-3L) for the two treatment alternatives; surgery or non-surgical treatment. The purpose of ERVIN.2 is to reduce the number of surgeries and provide a tool to reduce the number of patients who are offered surgery but have no clinical or HRQoL benefits and vice versa, to reduce the number of patients who are not offered surgery but would benefit from it.

A national clinical coordination group of surgeons, physiotherapists, patients, nurses and other experts recommended these outcome measures as usable patient-reported outcome measures for baseline and 1-year follow-up scores [15, 16]. The rationale for using Oxford scores for collecting data is that they are designed to assess function and pain with patients undergoing hip replacement surgery and best validated in a Danish context [17]. ERVIN.2 does not allow for more than one clinical PRO questionnaire to avoid unreliable answers.

The scores are presented in a simple graphical interface, which can be included in the interaction between the patient and the surgeon during consultations. By comparing usual care and an ERVIN.2-based care model, the study aims to investigate if ERVIN.2 enhances clinical decision-making by increasing patient involvement (measured as SDM-Q9 and SDM-doc) without reducing the health (measured as OHS or OKS) of the patients. Therefore, a non-inferiority design is chosen. Furthermore, the aim of the economic evaluation carried out alongside the RCT is to evaluate whether ERVIN.2 is cost-effective compared to usual care. The objective of the present trial protocol is to provide the study design and planned analyses for the RCT and subsequent economic evaluation.

The primary aim of this study is to determine if usual practice (control) is non-inferior to using ERVIN.2 as an add-on to usual practice (intervention) measured by OKS and OHS for patients with OA who are referred to the orthopaedic department to decide if they should have total hip or knee replacement surgery. Secondary aims are to determine the degree of shared decision-making (SDM) by comparing the control and intervention groups as well as conducting a full health economic evaluation.

## Methods

### Trial design

A single-centre, patient-blinded 1:1 randomised-block controlled two-arm parallel-group non-inferiority trial was conducted from 30.11.2021 to 31.12.2022.

### Study setting

A single-site trial will be carried out at the Department of Orthopaedic Surgery at Aalborg University Hospital, Farsoe. The department performs orthopaedic surgery on patients with OA in their hips and knees. The clinic receives 3000 new referrals for THR or TKR consultations per year, and of these, 1000 patients have joint replacement surgery. The clinic has eight permanent hip and knee specialist surgeons and a varying number of younger doctors in training programmes. The patients are referred by a general practitioner (GP) to the clinic to consult a surgeon about total hip or knee replacement following OA. Only permanent surgeons are included in the trial.

### Eligibility criteria

Patients will receive the first trial information at home by secure e-mail along with an invitation to the clinical assessment. When the patient arrives at the clinic, a designated helper will show him or her to the electronic PRO (ePRO) kiosk and orally inform the patient about the trial. Patients who agree to participate in the trial will sign the informed consent when logging into the ERVIN.2 system. The designated helper will be available in the room when the ePRO is filled out, should any doubts or questions arise. If the patient declines to participate in the trial, their name is registered in a log. If a participant withdraws informed consent, data are not included for analyses or further collection. Trial information and obtainment of informed consent are collected and stored in agreement with Danish legislation. No biological specimens were involved in the study.

## Interventions

### Treatment as usual

The conventional approach to decide if hip or knee replacement should be chosen includes a 30-min consultation in which the surgeon evaluates the need for surgery. This decision is based on the patient’s general health and subjective description of pain and disabilities. Additional health information from the referring GP may also be assessed. The orthopaedic surgeon evaluates x-rays and eventual MRI or CT scans and conducts a comprehensive physical examination focusing on the lower extremities and lower back. The findings and knowledge obtained are discussed with the patient before the decision is made.

### Intervention group

The justification of ERVIN.2 as a comparator is its ability to capture and estimate the baseline and predicted clinical outcomes for OHS or OKS and expected HRQoL for the non-surgical treatment or surgical choice. During the consultation, only the intervention group will have the results from ERVIN.2 as an interface with a graphical presentation of real-time and predicted outcome scores (OHS or OKS and EQ-5D-3L).

**Fig. 1:**
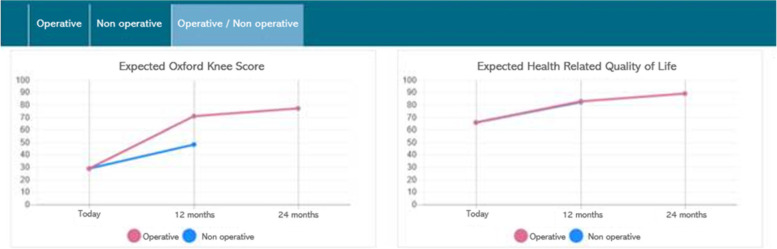
Graphical presentation of baseline (real-time) and predicted OKS and EQ-5D-3L score. The *x*-axis = time in years, and the *y*-axis = normalised score scale). The 1- and 2-year predictive scores are shown for the operative choice (red), and 1-year prediction scores for the non-operative choice (blue)

This trial builds upon the experiences from using ERVIN.1, which can predict outcomes for surgical choices. The development of ERVIN.1 began in 2013, and the system has been available in the department since 2018. However, its usage is unknown since it is not a part of the usual clinical routine. In 2020, ERVIN.2 replaced ERVIN.1, and this version can predict outcome scores for both surgical and non-surgical choices. The learning datasets used for development and training for ERVIN.1 and ERIN.2 do not differ from the study centre except for the time difference. All hip and knee patients included in the current trial enter data in the ePRO kiosk, including OHS, OKS, and EQ-5D-3L questionnaires. ERVIN.2 combines the ePRO data with data merged from the patient administrative system (PAS) and the electronic laboratory system (LABKA). The merged dataset is then applied in real-time predictive analyses, estimating expected outcome scores for the individual patient [18]. ERVIN.2 then visualises the ePRO data in a shared interface, which shows normalised real-time baseline values and 1-year predictive scores for the expected change in OKS or OHS, ranging from 0 to 100, and EQ-5D-3L scores ranging from 1 to 100 (Fig. 1).

The trial applies a revised version of the prediction algorithm in ERVIN.2, completed by the end of 2020 (publication in progress), which allows for prediction scores related to the conventional treatment. Until now, there is no evidence about the effects of the AI-based decision support system ERVIN.2. However, an evaluation report of ERVIN.1 has confirmed the feasibility and pointed to a large potential benefit for patients and doctors [19]. All intervention surgeons have a one-to-one training session with the project surgeon at the department where the new features are explained and tested before the trial begins. Subsequently, a pilot period of approximately one month was included to adjust for the learning curve. The trial description and flowchart are available in all consultation rooms.

In the inclusion period, surgeons are obligated to present the ERVIN.2 interface during the consultation to those allocated to the intervention group. The intervention is designed to guide the decision on surgery, and the decision will not affect the subsequent treatment. Thus, further attempts to modify or increase adherence to treatment will not be made.

### Outcomes

It is unclear if there is a clinically important improvement in the 6- to 12-month recovery period after hip and knee replacement [20]. Some evidence was found in a systematic review that clinically important improvement in the OHS and OKS occurred in the 6- to 12-month recovery period. It recommended using 12- rather than 6-month outcome data [20]. The 1-year follow-up time horizon was also discussed and accepted as appropriate by dedicated orthopaedic specialists for both THR and TKR. Thus, follow-up data for OKS, OHS and EQ-5D-3L in this study is collected 1-year post baseline (first consultation) for non-surgical patients and 1-year post-surgery for patients choosing surgery. These outcome measures and time horizon are also adopted by the steering group of The Danish Clinical Quality Program and National Clinical Registries for hip and knee OA [15].

#### Primary outcome

The primary outcome measures are OHS and OKS, depending on the indication. Both questionnaires are developed to assess pain and function for patients undergoing hip or knee replacement surgery and are available in Danish-validated versions [21, 22]. The questionnaires are short, 12-item, patient-reported outcome (PRO) questionnaires, including two domains: pain and function with six items each. The questionnaires are reported to be valid, reproducible and sensitive to clinically important changes [23]. Item scores are summed to give a total score between 0 and 48, with higher scores indicating a better outcome [23, 24].

#### Secondary outcomes

To establish if ERVIN.2 enhances clinical decision support the SDM-Q9 and SDM-Q-doc questionnaires are used, as these measure the extent to which patients are involved in decision-making from a patient perspective (SDM-Q9) and a physician perspective (SDM-Q-Doc) [25]. Both versions are applicable in preference-sensitive decisions where several treatment options exist for a particular disease [25]. The questionnaire comprises nine items and six levels. Item scores are summed to a total score between 0 and 45. The higher the score, the better the outcome [26]. SDM-Q9 is translated and validated in a Danish setting, and the validation of SDM-Q-Doc is in progress [25, 27]. SDM-Q9 and SDM-doc questionnaires are completed once immediately after the first consultation but in a separate room from the consulting surgeon. The EQ-5D-3L measures HRQoL, which is used to estimate the quality-adjusted life years (QALY), the gold standard for health economic evaluations [28]. EQ-5D-3L is available in a Danish digital version, and Danish preference weights are used to estimate utility scores [29, 30]. The questionnaire comprises five dimensions: mobility, self-care, usual activities, pain/discomfort, and anxiety/depression. Each dimension has three levels. Item scores are estimated to be a total score between ±1, where 1 equals perfect health, 0 equals dead, and negative scores equal worse than dead [31]. EQ-5D-3L data is collected concurrently with the OHS or OKS questionnaire (Table 1).

**Table 1:** Summary of primary and secondary outcomes, questionnaire abbreviations and time for data collection

Domain for measurement	Name	Timeline for data collection
Function and pain	Oxford Hip Score (OHS)	Baseline and 1-year follow-up
Function and pain	Oxford Knee Score (OKS)	Baseline and 1-year follow-up
Health-Related Quality of Life	EQ-5D-3L	Baseline and 1-year follow-up
Shared Decision Making	SDM-Q9	Baseline
Shared Decision Making	SDM-Q-Doc	Baseline

### Participant timeline

**Fig. 2:**
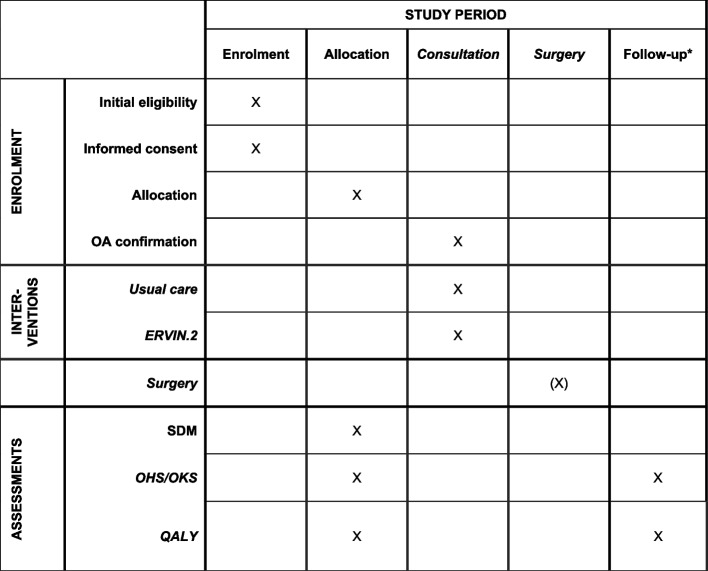
Schedule of enrolment, interventions, and assessments. *1-year post-consultation (baseline) for non-surgical patients and 1-year post-surgery for surgical patients

Patients are enrolled in connection with the first consultation in the clinic and allocated directly after informed consent is given and OHS or OKS and EQ-5D-3L questionnaires are filled in. Patients who, after the first consultation, are not regarded as OA patients and have been wrongly referred to the clinic are excluded. The SDM questionnaires are filled in immediately after the consultation at baseline. Follow-up data are collected one-year post-surgery or 1-year post-consultation for surgical patients and non-surgical patients, respectively (Fig. 2).

### Non-inferiority margin

To demonstrate non-inferiority a four points margin is used for both the OHS and OKS scales. Four points are half the size of what is considered to be a clinically relevant difference from the patient perspective [32].

### Sample size

The sample size is estimated with a 5% significance level, 90% power, 10% drop-out, and an assumed standard deviation of change equal to 10.4 points, which also corresponds to the four-point non-inferiority margin. We arrive at an estimated sample size of 130 subjects in each treatment arm. We did not account for a randomised block design in our calculation, resulting in a possibly larger sample size than necessary [30].

### Recruitment, randomisation and blinding

Trial participants are recruited through the orthopaedic outpatient clinic at Aalborg University Hospital. Patients will be randomised to either control or intervention using a randomised block design stratified by sex, age, OHS or OKS baseline scores and location of disability (hip or knee). Age (≤ 60, ≥ 61) and Oxford scores (0–40, 40–100) are subdivided into two categories. Randomisation is done using REDCap electronic data capture [33]. Block sizes are random, with possible volumes of two and four patients. Patients are randomised following baseline questionnaire completion (OHS or OKS and EQ-5D-3L) at the ePRO kiosk. The designated helper enters information on randomisation into a separate REDCap module. A randomisation key is then automatically generated, which can be extracted by the designated helper but is blinded from the patient. After randomisation, the patient receives a blue intervention group envelope or a yellow control group envelope containing the SDM-Q9 and SDM-doc questionnaires, which he or she brings to the following consultation. The randomisation key is blinded from the investigators throughout the trial and only accessible by the data monitoring committee and the treating surgeon.

Blinding of the consulting surgeon is not possible since the intervention is an interface add-on to the conventional treatment. Blinding of patients means that patients do not know that ERVIN.2 is the intervention. After trial completion, data is stored in a safe data storage with transcription logging awaiting further analysis, at which point the randomisation key is revealed to the investigators.

## Data collection and management

OHS, OKS and EQ-5D-3L data are collected through ERVIN.2, which constitutes the Case Report Form from which the data will be exported and analysed. The IT infrastructure and integrated data registers used in the it-system and ERVIN are described in Bjerregaard et al. [18]. SDM-Q9 and SDM-Doc are handed out as physical questionnaires stored in concordance with Danish legislation. Physical questionnaires are registered in Microsoft Excel by two independent persons and cross-controlled to reduce the risk of typing errors after full enrolment of patients.

The collection of follow-up data will be initiated one year from the baseline. Patients receive an OHS or OKS questionnaire and the EQ-5D-3L questionnaire electronically by secure e-mail 14, 7 and 3 days before the 1-year follow-up. Patients who do not answer on the day of 1-year follow-up will be contacted by a designated helper to fill in the questionnaires by phone.

## Statistical analysis

The primary analysis is conducted using mixed-effects linear regression with a random effect on the stratification groups. Similarly, secondary analyses are conducted using mixed-effects Poisson and linear regression for outcomes to obtain relative risks and risk differences as well as linear regression for continuous outcomes [34]. All analyses are performed in Stata 17 (StataCorp. 2021. Stata Statistical Software: Release 17. College Station, TX: StataCorp LLC).

A health economic evaluation will be conducted comparing ERVIN.2 as an add-on to the conventional consultation, following international guidelines for economic evaluations. The base-case analysis will include direct and overhead costs estimated as a mixed micro- and gross costs approach from a hospital perspective. Costs will be reported in euros (2022 index value). The measure of effect will be the gold standard for health economic evaluations, QALY, based on estimates from the EQ-5D-3L questionnaire and Danish population utility weights [30, 35]. A 1-year time horizon will be used, thus cost and effects will not be discounted. Results will be presented as an incremental cost-effectiveness ratio, plotted in an incremental cost-effectiveness (ICE)-plane to determine the cost-effectiveness of ERVIN.2 [36]. Threshold values of cost-effectiveness will only be considered if relevant, according to ICE-plane placement of the ICER. To investigate the robustness of the evaluation, relevant deterministic sensitivity analyses will be conducted based on a tornado diagram to heighten the understanding of cost and effect drivers. If necessary, additional analyses will be included, depending on the findings. In addition, probabilistic sensitivity analysis will be used to assess joint parameter uncertainty presented in an ICE scatterplot and a cost-effectiveness acceptability curve [36].

All analyses will be conducted according to intention-to-treat. However, patients who are lost to follow-up will be excluded from the base case analyses and included in sensitivity analyses, using multiple imputations and adjusting for relevant baseline differences between complete cases and patients lost to follow-up.

### Interim analyses

As the inclusion period is relatively short compared to the follow-up period, no interim analyses are planned.

## Data monitoring

Data monitoring is continuous to ensure that data quality is sufficient for subsequent statistical analyses. The monitoring task is performed by one person who is not blinded from the randomisation and not involved in the trial during the trial period. The purpose of the monitoring is to minimise missingness and ensure the quality of the collected data. The committee will monitor the baseline data when 10% of the participants are enrolled and the follow-up data when 10% of the participants have been concluded. Additional monitoring will only be conducted if the committee finds it necessary during the trial.

The intervention is an add-on to the conventional treatment, expressed as an interface with a graphical transformation of real-time and predicted ePRO data. The intervention is considered risk-free, confirmed in the feasibility study [37].

## Dissemination plans

The trial results will be published in international medical and health economic journals with peer review. Findings over and under the margin of non-inferiority, as well as inconclusive findings, will be published.

## Discussion

In this study, we expect no difference in OHS or OKS when using ERVIN.2 as an add-on to usual practice. A study by De Achaval et al. showed potential to improve SDM in patients with knee osteoarthritis, considering TKR measured by using the decisional conflict scale developed by O’Connor. However, the association with changes in the Knee Injury and Osteoarthritis Outcome Score (KOOS) was not covered [38]. Another study achieved inconclusive results when investigating the effect of enhanced SDM on clinical outcome (KOOS or Harris hip score) for patients who were to decide on joint replacement surgery or not [39]. The findings showed a positive impact on knee patients but a negative impact on hip patients. The interventions in the two studies did not use advanced technology such as AI-based software to improve clinical and decision support in orthopaedic surgery [40].

The design of this study does not allow for the blinding of surgeons to reduce bias. The development of ERVIN.1/.2 was based only on data from a single centre, which constituted a limitation concerning generalisability. However, the Danish healthcare system is tax-financed and free for all citizens, and the national quality of THR and TKR is of high quality. Another limitation is that the non-surgical patients can choose different treatment courses after the consultation in the outpatient clinic. The results from this study seek to increase knowledge about ERVIN.2 and whether the use of the clinical decision support systems is associated with an increased sense of SDM, while clinical outcomes in the form of OHS or OKS and QALY remain unchanged. In addition, we seek to investigate whether ERVIN.2 is cost-effective. The results can guide decision-makers by providing transparency about potential health-related benefits for patients and efficient allocation of scarce healthcare resources.

## Trial status

At the time this manuscript was completed, 300 patients were recruited from 30.11.2020 to 12.03.2021. The final results will be completed by approximately 30.06.2023.

## Data Availability

Study data can be retrieved if requests fulfil all requirements, according to Danish legislation.

## Abbreviations

AI: Artificial intelligence
CDSS: Clinical Decision Support System
CONSORT: Consolidated Standards of Reporting Trials
ePRO: Electronic patient-reported outcomes
GP: General practitioner
HRQoL: Health-related quality of life
ICE: Incremental cost-effectiveness
KOOS: Knee injury and Osteoarthritis Outcome Score (KOOS)
LABKA: Electronic laboratory system
OA: Osteoarthritis
OHS: Oxford Hip Score
OKS: Oxford Knee Score
PAS: Patient administrative system
PRO: Patient-reported outcomes
QALY: Quality-adjusted life years
RCT: Randomised controlled trial
REDCap: Research electronic data capture
SDM: Shared Decision Making
THR: Total hip replacement
TKR: Total knee replacement
